# Spontaneous Clearance of a Secondary Buruli Ulcer Lesion Emerging Ten Months after Completion of Chemotherapy—A Case Report from Togo

**DOI:** 10.1371/journal.pntd.0001747

**Published:** 2012-07-31

**Authors:** Marcus Beissner, Ebekalisai Piten, Issaka Maman, Dominik Symank, Moritz Jansson, Jörg Nitschke, Komi Amekuse, Basil Kobara, Franz Wiedemann, Harald Hoffmann, Adolf Diefenhardt, Kossi Badziklou, Abiba Banla Kere, Thomas Löscher, Gisela Bretzel

**Affiliations:** 1 Department of Infectious Diseases and Tropical Medicine (DITM), University Hospital, Ludwig-Maximilians University, Munich, Germany; 2 Centre Hospitalier Régional Maritime (CHR Maritime), Tsévié, Togo; 3 Institut National d'Hygiène (INH), Lomé, Togo; 4 German Leprosy and Tuberculosis Relief Association (DAHW), Würzburg, Germany; 5 German Leprosy and Tuberculosis Relief Association (DAHW), Togo office, Lomé, Togo; 6 Programme National de Lutte contre L'Ulcère de Buruli – Lèpre et Pian (PNLUB-LP), Lomé, Togo; 7 Institute of Microbiology and Laboratory Medicine, Pneumological Teaching Hospital of the University of Munich, Gauting, Germany; Kwame Nkrumah University of Science and Technology (KNUST) School of Medical Sciences, Ghana

## Presentation of Case

**Figure 1:**
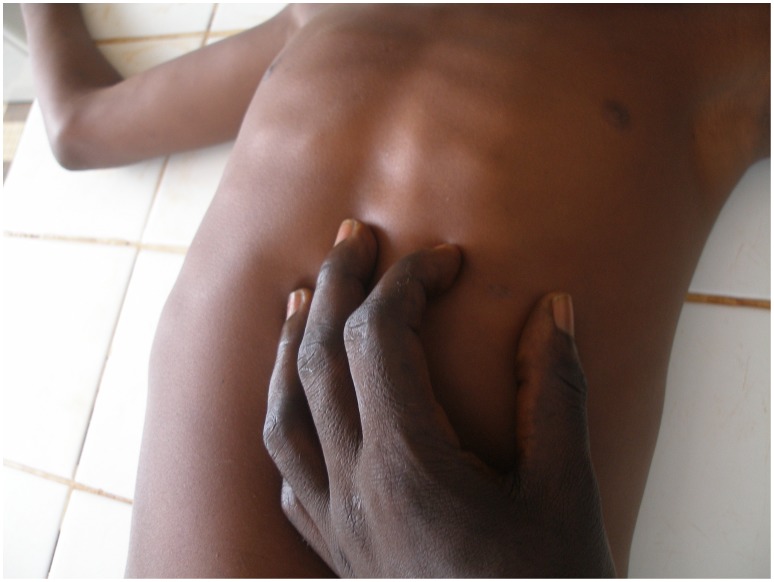
Primary nodule at the left costal arch, June 2010.

**Figure 2:**
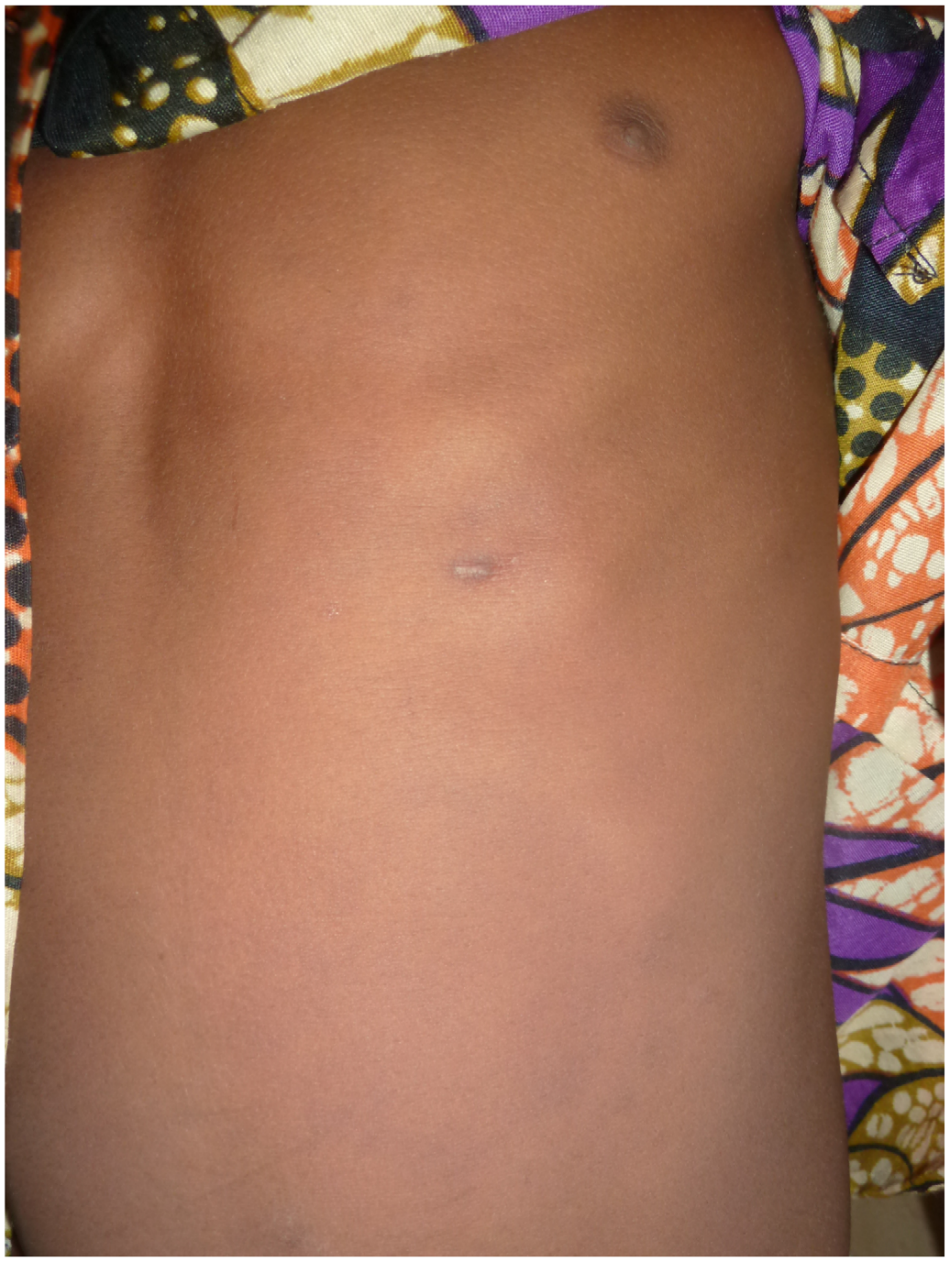
Scar of the primary nodule at the left costal arch, September 2010.

**Figure 3:**
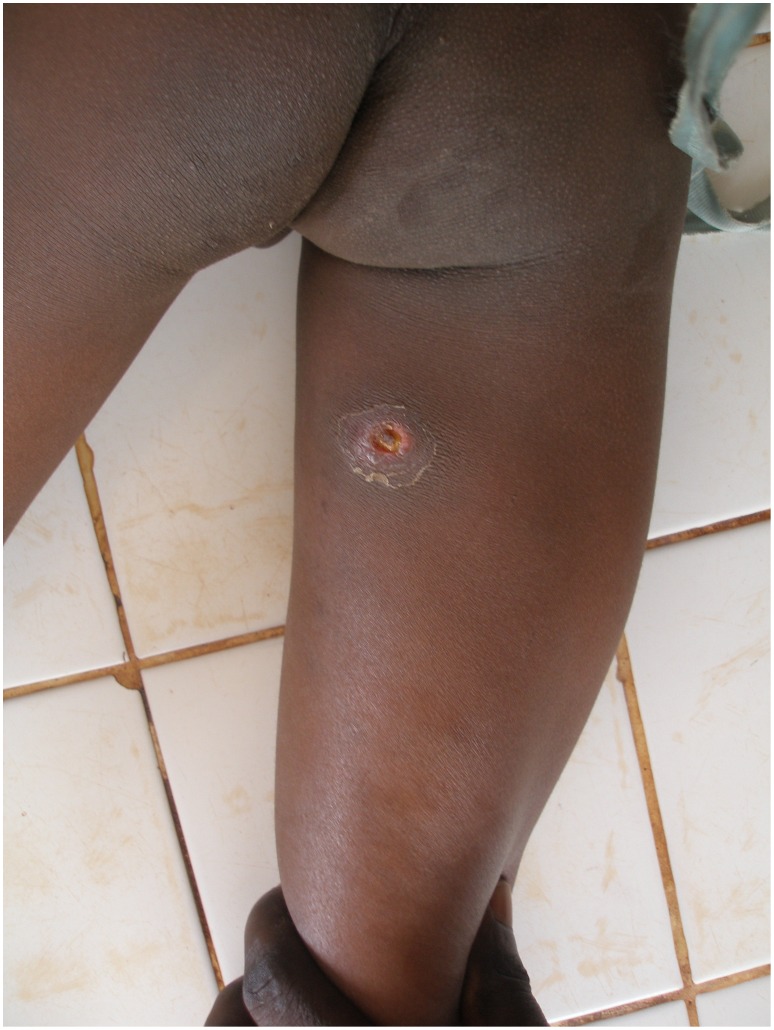
Secondary ulcerated nodule at the back of the right thigh, July 2011.

**Figure 4:**
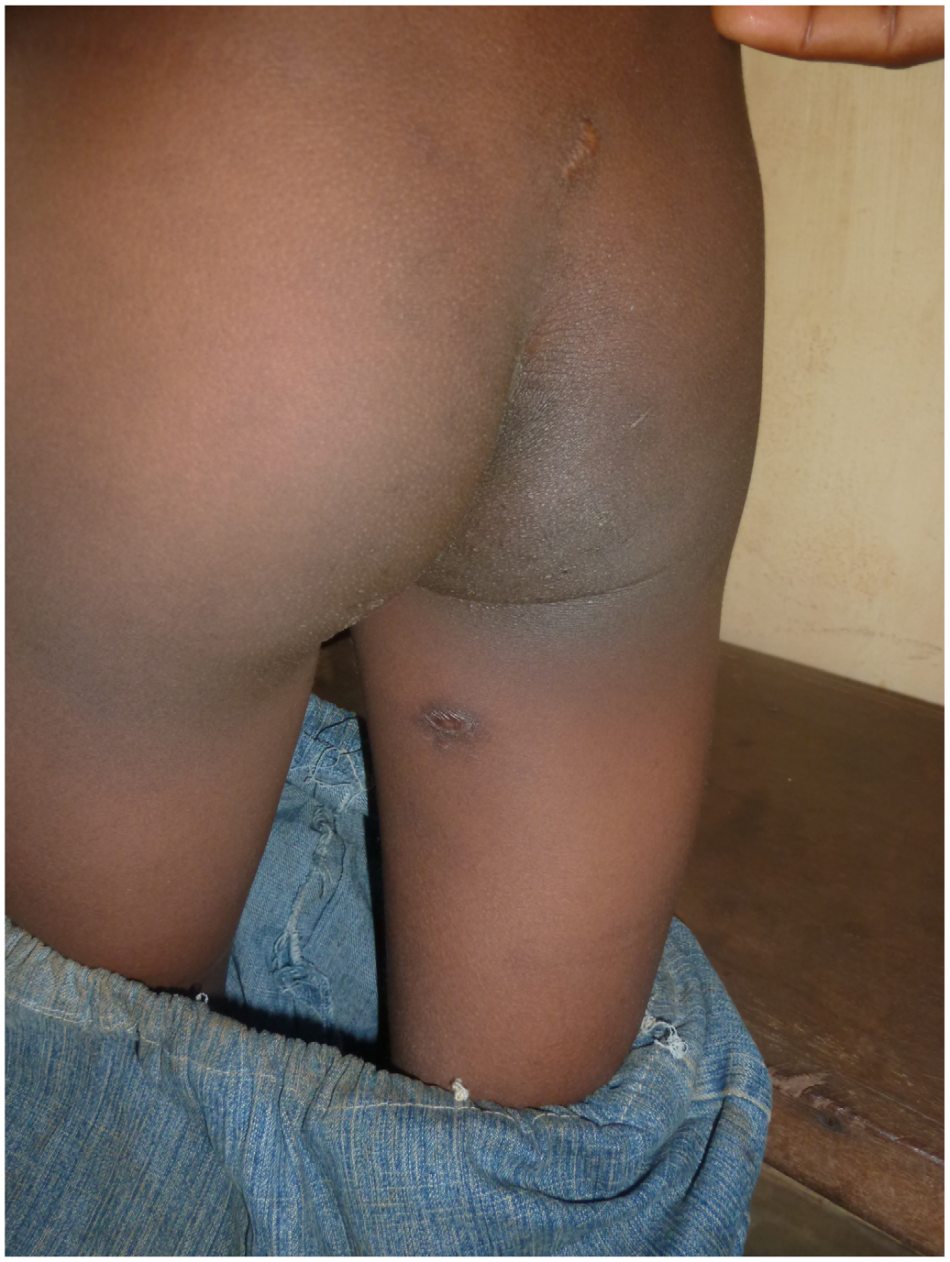
Scar of secondary nodule at the back of the right thigh, September 2011.

An eight-year-old boy from Togo presented with a nodule of 30 mm in diameter at the left costal arch (Figure 1) clinically compatible with Buruli ulcer disease (BUD) at the “Centre Hospitalier Régional Maritime” (CHR Maritime), Tsévié, in July 2010. His hometown, a village located close to the river “Haho” in the central district “Yoto” of the “Région Maritime”, constitutes one of the BUD-endemic foci in Togo [1]. The patient's BCG vaccination status was positive and no other family member was diagnosed with BUD before. The lesion was laboratory confirmed by conventional IS*2404* PCR from a 3-mm punch biopsy sample (Table 1) at the Department of Infectious Diseases and Tropical Medicine (DITM), Munich, and a full course of rifampicin (300 mg/d) and streptomycin (0.5 g/d) was administered for eight weeks at the peripheral health post (“Unité de Soins Périphérique”, USP). The patient was fully compliant throughout the entire period of treatment and no complications were reported. In September 2010, the lesion was completely healed under scarification (Figure 2) and the patient was considered cured. Weekly follow-up was conducted by the BUD nurse of the village for three months following healing accompanied by monthly follow-up by the BUD nurse of CHR Maritime. No pathological findings were observed until June 2011. In July 2011, the boy (in the meantime nine-year-old) presented again with a secondary nodule (diameter: 30 mm) at the back of the right thigh (Figure 3) at CHR Maritime one week after its emergence. The lesion was clinically compatible with BUD and clinical samples were collected and forwarded to the newly established BUD Reference Laboratory at the “Institut National d'Hygiène” (INH), Lomé, as well as DITM (Table 1). Microscopy of a Ziehl-Neelsen stained FNA smear was (scanty) positive and the presence of *Mycobacterium ulcerans* DNA was confirmed by IS*2404* real-time qPCR at DITM while conventional IS*2404* PCR remained negative for all samples tested. The secondary lesion ulcerated three weeks after emergence and further samples were collected from the ulcer (diameter: 25×30 mm) and forwarded to DITM for analysis. Whereas IS*2404* qPCR reconfirmed the presence of *M. ulcerans* DNA, viability testing of *M. ulcerans* by analysis of mycobacterial ribosomal 16S RNA through a newly established 16S rRNA RT qPCR (specificity: 100%, positivity rate for pre-treatment swab samples: 83.3% [95%-CI: 66.1%–100%], limit of detection: six copies of the target sequence) (unpublished data) and culture were negative (Table 1). Under stringent clinical observation, conventional wound care by daily cleaning with normal saline, disinfection with povidone-iodine, and sterile dressing of the ulcerated lesion was performed at the USP for two weeks. The lesion healed completely (Figure 4) five weeks after onset of disease. The patient's parents gave written informed consent for publication.

**Table 1:** Clinical samples and laboratory results of primary and secondary BUD lesions.

Date of Sample Collection	Clinical Presentation and Diameter of Lesion	Sample Type	Transport Medium	Laboratory Results				
				MIC^a^	IS*2404* PCR^b^	IS*2404* qPCR^c^	16S RT qPCR^d^	CUL^e^
July 20, 2010	Primary nodule, 30 mm	FNA^f^Punch biopsy	CLS^g^CLS	negND	negpos	ND^h^ND	NDND	NDND
July 13, 2011	Secondary nodule, 30 mm	FNA(1)FNA(2)	CLSPANTA^j^	pos (1AFB^i^)neg	negpos	posneg	NDND	NDND
July 21, 2011	Ulcerated secondary nodule, 25×30 mm	Punch biopsySwab (1)Swab (2)Swab (3)	PANTAPANTAPANTAPANTA	negnegNDND	NDNDnegneg	NDNDpospos	NDNDnegneg	No growth^k^No growthNDND

Table 1 shows samples collected from the primary and secondary lesions in July 2010 and 2011 and the corresponding laboratory results. “Neg” indicates a negative test result, “pos” indicates a positive test result.

a MIC, microscopic examination of acid fast bacilli (AFB) following Ziehl-Neelsen staining conducted at CHR, DITM, and INH (samples from secondary lesion only).

b IS*2404* PCR, conventional, single-step, gel-based IS*2404* polymerase-chain-reaction conducted at DITM.

c IS*2404* qPCR, real-time quantitative IS*2404* polymerase-chain-reaction conducted at DITM.

d 16S RT qPCR, *Mycobacterium ulcerans*–specific reverse-transcription real-time quantitative polymerase-chain-reaction targeting the ribosomal 16S RNA of *M. ulcerans* conducted at DITM.

e CUL, mycobacterial culture on Löwenstein-Jensen medium conducted at IML red, synlab, Asklepios Gauting, Germany.

f FNA, fine-needle aspiration.

g CLS, Puregene cell lysis solution, Qiagen, Germany.

h ND, not done.

i AFB, acid fast bacilli.

j PANTA, transport medium for viable mycobacteria containing Polymyxin B, Amphotericin, Nalidixic acid, Trimethoprim, and Azlocillin.

k No growth, culture result negative, no growth of acid fast bacilli.

## Case Discussion

BUD caused by infection with *M. ulcerans* may lead to extensive destruction of the skin, soft tissue, and bone with severe fibrous scarring and formation of contractures if left untreated. Pathogenesis of BUD is mediated by the cytotoxic and immunosuppressive exotoxin mycolactone [2]. During the last decade, significant advances in the treatment of BUD have been made and the introduction of standardized antimycobacterial chemotherapy with rifampicin and streptomycin resulted in recurrence rates below 2% [3].

While recurrences after surgical excision alone presumably are attributable to the persistence of mycobacteria in macroscopically healthy tissue bordering surgical excision [4], little is known about the pathogenesis and immunological mechanisms of secondary BUD lesions evolving after completion of standardized antimycobacterial treatment [5].

Development of new skin lesions during antimycobacterial treatment are currently assumed to be caused by immune-mediated, paradoxical reactions (i.e., deteriorating responses to treatment of an infection after initial improvement) which are likely to be triggered by mycobacterial antigens and immune-stimulators released from killed mycobacteria [6], [7].

Ruf et al. recently reported two BUD patients from Benin who developed a series of secondary BUD lesions after completion of chemotherapy [5]. These lesions may partly represent secondary infection foci that were already present during treatment and appeared as a consequence of delayed paradoxical reactions. However, in particular, lesions occurring more than one year after completion of treatment may have been associated with new *M. ulcerans* infection or mycobacteria surviving antimycobacterial treatment and may have been resolved by immune responses triggered by successful treatment of primary lesions.

In accordance with the other cases published so far, in the present case a secondary *M. ulcerans* lesion was laboratory confirmed by microscopic detection of acid fast bacilli and IS*2404* real-time qPCR, whereas cultures remained negative. Furthermore, analysis of mycobacterial ribosomal 16S RNA did not provide evidence for the presence of viable bacilli.

As shown by Ruf et al. histopathological analysis of surgically excised late-onset secondary lesions revealed characteristical features of BUD as well as massive leukocyte infiltration of necrotic areas characteristic for successfully treated lesions. As there was no surgical intervention for the secondary lesion of the Togolese patient, clinical samples for histopathological analysis were not available.

Pathogenesis of the secondary BUD lesion in the present case might either be attributable to a second unrecognized focus of killed *M. ulcerans* during antibiotic chemotherapy ten months earlier which became clinically apparent due to a late inflammatory response to residual mycobacterial antigens (i.e., late paradoxical reaction), or to re-inoculation of *M. ulcerans* that was cleared by an elevated immune response primed by the successful initial treatment. However, available laboratory methods did not allow distinguishing between late paradoxical reaction and spontaneous host clearance during a second exposure.

While mycolactone plays a major role in the pathogenesis of primary BUD lesions, the question whether and to which extent the toxin is involved in the pathogenesis of secondary BUD lesions remains unresolved. Sarfo et al. recently demonstrated the detection of mycolactone in human tissue, suggesting its usefulness as a biomarker for monitoring the clinical response to treatment [8]. Detection of mycolactone in secondary lesions may support the hypothesis that new infection foci are associated with secondary lesions. However, to our knowledge, data on mycolactone in secondary lesions are still lacking.

Beside previous anecdotal observations on spontaneous clearance of lesions in clinically suspected BUD cases, Gordon et al. recently reported the first case of spontaneous resolution of a laboratory confirmed BUD ulcer in a patient from Australia [9]. Whereas the secondary lesions of the two BUD patients from Benin were surgically excised, the ulcerated lesion of the Togolese case also healed under conventional wound care.

In the absence of evidence-based guidelines for reliable identification of late-onset secondary immune-mediated lesions and their clinical management, it may be advisable to consider the possibility of spontaneous healing under stringent clinical observation and regular wound care.

**Learning Points**

- Secondary BUD lesions may occur as paradoxical reaction (i.e., deteriorating responses to treatment of an infection after initial improvement) during or shortly after treatment; late-onset secondary lesions may occur up to more than one year after completion of treatment.
- Characteristic diagnostic results for secondary BUD lesions are positive microscopy and PCR results without evidence for viable bacilli.
- The case of the Togolese patient shows that complete healing of secondary lesions without antibiotic or surgical treatment occurs. Therefore, conventional wound care can be considered as a treatment option if continuous clinical observation is possible.
